# ‘I'm willing to walk into violence’: The impact of personal trauma on staff compassion in secure services

**DOI:** 10.1111/papt.70030

**Published:** 2025-12-12

**Authors:** Bethan Owen, Tobyn Bell, Katherine Berry, Brendan J. Dunlop

**Affiliations:** ^1^ Greater Manchester Mental Health, School of Health Sciences, The University of Manchester Manchester UK; ^2^ Division of Psychology and Mental Health, School of Health Sciences, Faculty of Biology, Medicine and Health The University of Manchester Manchester UK

**Keywords:** compassion, forensic mental health, qualitative, staff well‐being, trauma

## Abstract

**Objectives:**

Current evidence on the impact of personal trauma on health care professionals is mixed. Some studies suggest a personal history of trauma can be a risk factor for secondary traumatisation. Other research suggests that personal experiences of trauma can help professionals to better recognise their patients' trauma symptoms. Staff working in forensic mental health services face challenges, such as exposure to patient self‐harm and suicide. This study aimed to understand the impact of personal trauma on staff working in forensic mental health services and how this impacts their ability to give compassionate care in a challenging environment.

**Design:**

Qualitative methodology was used. Participant interviews were analysed using Interpretative Phenomenological Analysis.

**Methods:**

Seven participants were recruited from forensic mental health services across the Northwest of England who had personal histories of trauma. Participants completed a semi‐structured interview and questionnaires recording traumatic life experiences. Interviews focused on how staff were impacted by their experiences of trauma and if this had an impact on them giving compassionate care.

**Results:**

Three main themes were identified from participant interviews: reliving the past in the present, true empathy as both a gift and a burden, and the costs and benefits of gaining personal meaning and being changed by the work.

**Conclusions:**

Participants reported that trauma had both positive and negative impacts on their work. Overall, a common thread that emerged in all interviews was that trauma did not appear to be a barrier to compassion but rather a facilitator.

## INTRODUCTION

The psychological impact of trauma can have a number of negative and uncontrollable effects on a person's life that may persist long after the event. This can be emotional, physical, cognitive, behavioural, social and developmental (Center for Substance Abuse Treatment, 2014). The impact of trauma in health care professionals (HCP) is of particular interest as they are at higher risk of being exposed to traumatic experiences at work due to the nature of the jobs compared with other professions (Brower & Riba, 2017; Flannery, 2015; Spaan et al., 2024).

The influence that trauma can have on HCPs has the potential to impact patient care as well as the mental health of HCPs. For example, previous research has found a negative relationship between experiences of workplace bullying and the quality of patient care (Purpora et al., 2015). Furthermore, nurses who were interviewed after experiencing violence from patients reported it left them feeling more vigilant when caring for other patients; an effect that was associated with lower levels of self‐reported resilience within staff (Hollywood & Phillips, 2020). There is evidence to suggest that mental health professionals' own trauma experienced outside of the work environment can increase the likelihood of secondary trauma stress from exposure to workplace trauma (Hensel et al., 2015), which in turn may negatively impact patient care through a reduction in empathy (Lakioti et al., 2020), highlighting the need to mitigate the impacts of staff trauma in order to enhance patient care.

Compassion is defined as a sensitivity to suffering in oneself and others, accompanied by a commitment to alleviate and prevent it (Gilbert & Choden, 2013) and is considered a crucial component of health care (Sinclair et al., 2017). Compassion is defined as a motivation which is closely associated with caregiving and can be differentiated from emotions and competencies, such as empathy, which might be recruited for other motives (Gilbert, 2020). Compassion is experienced in three distinct ‘flows of compassion’; these include feeling compassion towards others, feeling compassion from others and compassion towards oneself (Gilbert, 2009, 2010). Research suggests that patients who experience compassionate care have better treatment outcomes than those who do not (Lown et al., 2015). Furthermore, higher levels of job satisfaction are linked to HCPs providing compassionate care (Sinclair et al., 2016). Unfortunately, burnout and compassion fatigue are common among HCPs (De Hert, 2020; Jin et al., 2021). Compassion fatigue has been defined as a health care practitioner's diminished capacity to care for others as a consequence of repeated exposure to patients' suffering (Cavanagh et al., 2020). However, there is some debate about the concept of compassion fatigue, and Gilbert (2017) has argued that it is not the motivation to care that is fatigued, but rather that health care staff can become burned out by excessive expectations and limited resources. However, compassion fatigue, as typically defined, can have negative physical effects (e.g. headaches, disturbed sleep and digestive issues) and emotional consequences (mood swings, anxiety and depression) for HCPs, as well as affecting patient care through decreased productivity and increased turnover (Lombardo & Eyre, 2011).

Staff working in forensic mental health services are more likely to be exposed to difficult situations, such as patient self‐harm and suicide, than in other health care settings (Stewart et al., 2012). Additionally, forensic services often face wider systemic issues, such as understaffing and a lack of resources (Wilson et al., 2018). Nurses working in forensic mental health reported being exposed to distressing events, such as violence, aggression and deliberate self‐harm (Mistry et al., 2022). Exposure to these events had several detrimental effects on nurses, including sleep disturbance, hypervigilance and self‐neglect (Mistry et al., 2022). Burnout and compassion fatigue are more common in HCPs working in forensic settings due to the intense nature of their work (Pirelli et al., 2020). Nurses working in forensic psychiatry reported struggling to remain empathetic and provide compassionate care (Sturzu et al., 2019). Part of this struggle could be due to staff feeling threatened and frightened by clients and their expressions of suffering (Hammarström et al., 2020).

Currently, the evidence on the impact of personal trauma on HCPs is somewhat mixed. For example, personal experience of trauma is a risk factor for secondary traumatisation for HCPs (Hubbard et al., 2017). Vicarious or secondary trauma is being traumatised as the result of being exposed to the trauma of others and has been associated with significant impairments in HCPs (Isobel & Thomas, 2022; Velasco et al., 2023). Furthermore, a personal history of trauma in therapists is associated with an increased risk of compassion fatigue (La Mott & Martin, 2019; Somoray et al., 2017). However, research suggests that when clients and clinicians are traumatised by the same events, clinicians are better able to recognise symptoms of trauma that may otherwise have been overlooked (Day et al., 2017).

Currently, there is minimal research on the impact of personal trauma on forensic mental health staff. Previous research on staff in forensic units has primarily recruited nurses exclusively. The current study will be open to all client‐facing staff in forensic settings. Therefore, it has the scope to potentially recruit a variety of professionals and broaden our current understanding of the impact of personal trauma on forensic mental health staff. Although there is research to show that a personal history of trauma can be associated with a negative impact on HCPs (La Mott & Martin, 2019; Somoray et al., 2017; Velasco et al., 2023), it is not yet understood how or why. In addition, we do not yet fully understand the impact of trauma on staff working specifically in forensic services, or how this may affect their ability to show or give compassion. This study aimed to make a novel contribution to the existing literature and give voice to an underrepresented population.

This study aimed to understand the impact of personal trauma on staff working in forensic/secure mental health services and how this impacts their ability to show compassion. This study aims to explore the following questions:

1. How do staff in forensic inpatient settings, with a personal history of trauma, experience giving care to their clients?
2. How do staff members' traumatic experiences impact them showing compassion in their professional role?
3. Do staff members experience any personal barriers or facilitators to the experience or demonstration of compassion for their clients?

## METHODS

### Design

This study aimed to understand the experiences of forensic mental health staff and how they made sense of these experiences. Therefore, this study utilised a qualitative design and interpretative phenomenological analysis (IPA). IPA is an approach designed for experiential research and has been informed by phenomenology, hermeneutics and idiography (Smith et al., 2022). IPA was chosen due to its idiographic nature; this allows the focus to remain on individuals' voices and the differences between their stories. IPA aims to explore lived experiences in detail rather than attempting to fit experiences into predefined categories. IPA is designed for in‐depth exploration of the lived experiences of a small group of people (Smith, 2004; Smith et al., 2009; Smith & Osborn, 2007), which is most compatible with the aims of this study.

### Recruitment and participant eligibility

Ethics approval was granted by the University of Manchester Research Ethics Committee 3. Additionally, this study was reviewed by the NHS Health Research Authority and approved by the Research and Development departments in the NHS trusts where participants were recruited. Participants were recruited from NHS adult forensic mental health services in the Northwest of England. The study was advertised using posters and via email distributed by management across adult secure services in two NHS trusts.

Participants were eligible to take part if they were over the age of 18, currently worked in a forensic/secure service in any patient‐facing role, could understand and speak English, consented to the interview being audio‐recorded and had self‐identified as having experienced a traumatic event at some point in their lifetime. In this study, trauma was defined using the following definition from the Diagnostic and Statistical Manual of Mental Disorders (2013):

Exposure to actual or threatened death, serious injury, or sexual violence in one (or more) of the following ways: Directly experiencing the traumatic event(s). Witnessing, in person, the event(s) as it occurred to others. Learning that the traumatic event(s) occurred to a close family member or close friend. In cases of actual or threatened death of a family member or friend, the event(s) must have been violent or accidental. Experiencing repeated or extreme exposure to aversive details of the traumatic event(s) (e.g., first responders collecting human remains; police officers repeatedly exposed to details of child abuse.)

This definition was shared with participants, and participants self‐identified as having experienced trauma using this definition.

### Measures

Participants were asked to complete two questionnaires. The first was a demographics questionnaire designed for the study, which captured details, such as age, job title, gender, sexuality and ethnicity. The second was the Life Events Checklist for DSM‐5 (Weathers et al., 2013), which is a checklist designed to identify potentially traumatic experiences across a person's lifetime. The demographics questionnaire was included to allow researchers to accurately describe the sample. The Life Events Checklist was used as a descriptive measure to characterise the experiences of the sample.

### Procedure

Participants were interviewed either over the phone or via video call by the first author. Although the research team would have preferred face‐to‐face interviews, a private, non‐clinical space was not always available. Additionally, participants expressed a preference for remote interviews due to their convenience. The interviews were semi‐structured, allowing participants to expand on the questions asked. Prior to the interview, participant consent was taken verbally and audio‐recorded separately. Interviews were conducted in a single session, lasting approximately 1 hour. After the interview, the interviewer checked on the participants' well‐being and provided them with a debrief letter, which signposted them to sources of psychological support.

### Data analysis

Participant interviews were audio‐recorded, transcribed and analysed using IPA methodology (Smith et al., 2022). In order to become immersed in the data, the first author transcribed each interview and analysed each individually before moving onto the next, in keeping with recommended IPA methodology and its commitment to idiography (Smith, 2011). IPA requires several stages of analysis: the researcher familiarising themselves with the material by reading and re‐reading the transcript, making exploratory notes, commenting on the raw data, constructing experiential statements based on the participants' own words (as well as exploratory notes) and grouping experiential statements to form emergent themes for each participant. After each participant's data were individually analysed, a group analysis was conducted, where individual themes were grouped together to create the final overall themes that reflect the experiences of the sample. During group analysis, the researcher referred to individual transcripts to consider how these themes manifested across the dataset.

### Rigour

To ensure the quality and rigour of data analysis, one of the researchers who was highly experienced in IPA methodology was consulted throughout the analysis process. The first author ensured the themes were grounded in the data at each stage by reviewing the raw data and exploratory notes in supervision. The third supervisor (TB) independently analysed half of the participant data, which was then compared and discussed with the first author. Final themes were created collaboratively in supervision using exploratory notes, personal experiential statements and cross‐referencing with the transcripts. Differences in analysis were resolved via negotiation and the inclusion of other team members for further triangulation.

### Reflexivity

It is well recognised in qualitative research that the views, standpoint and experiences of the researchers and research team can influence the analysis (Yardley, 2017). The first author was a Trainee Clinical Psychologist, and while they had worked with clients presenting with trauma histories, they had no previous experience working in or spending time in secure/forensic services. Therefore, the analysis could be completed from an ‘outsider’ perspective. Two of the authors (KB and BJD) were academics and consultant clinical psychologists with experience of working therapeutically in forensic settings. The third supervisor (TB) was a Cognitive Behavioural Psychotherapist and Compassion‐Focussed Therapist, with expertise in IPA. While all three supervisors used compassion‐focused therapy (CFT) in their clinical work, TB was an expert in CFT and frequently supervised and trained other practitioners in this approach.

The first author maintained a reflective log throughout the research process to identify and manage personal assumptions and expectations, and to monitor their impact. When such preconceptions were identified during analysis, the first author returned to the raw data to explore contradictions and exceptions. The reflective log was regularly discussed in supervision with the third supervisor (TB) to support reflexivity.

### Participant demographics

Seven participants completed the study, of whom four identified as female (57%) and three identified as male (43%). Participants' ages ranged from 20 to 42 years old. Six participants identified as White British (86%) and one identified as Asian/Asian British (14%). Four participants identified as bisexual (57%), two as heterosexual (29%) and one participant preferred not to provide details of their sexual orientation (14%). This study recruited participants from various job titles, including Occupational Therapist, Support Worker, Ward Administrator and Recovery Pathway Worker. Participants have been given pseudonyms, which have been intentionally not linked to demographic information to protect participant anonymity.

## RESULTS

### Traumatic experiences

Potentially traumatic experiences were recorded using Life Events Checklist‐5 (Weathers et al., 2013), and participant responses can be found in Table 1. The results from this questionnaire showed that participants had all been exposed to multiple distressing events.

**TABLE 1:** Results from Life Events Checklist‐5.

Event	Happened to me	Witnessed it	Learned about it	Part of my job	Not sure	Doesn't apply
Natural disaster	0	0	1	0	0	6
Fire or explosion	2	0	1	0	1	3
Transportation accident	2	1	1	0	1	2
Serious accident at work, home or during a recreational activity	0	2	0	0	0	5
Exposure to toxic substance	0	0	2	0	0	5
Physical assault	5	4	0	4	0	0
Assault with a weapon	2	1	1	1	0	3
Sexual assault	3	1	4	3	0	0
Other unwanted or uncomfortable sexual experience	3	1	3	2	0	2
Combat or exposure to war zone	0	0	1	1	0	5
Captivity	0	1	1	1	0	5
Life threatening illness or injury	2	1	3	0	0	3
Severe human suffering	1	1	2	4	0	2
Sudden violent death	0	1	1	0	1	4
Sudden accidental death	0	1	4	0	0	3
Serious injury, harm or death you caused to someone else	0	0	0	1	0	6
Any other very stressful experiences	2	2	1	2	2	2

### Themes

From the analysis of interviews came three interrelated but distinct superordinate themes. These are displayed in Table 2, below:

**TABLE 2:** Summary of themes.

Superordinate themes	Sub‐themes	Number of participants for each theme
Reliving the past in the present	Retraumatised and working with triggers	5
	Facing fears and rewriting the script	7
True empathy both as gift and burden	Lived experience as a tool	5
	Cost of empathy and connection	6
Identity: Giving and taking parts of yourself	The cost and benefit of giving your authentic self to your work	4
	Gaining personal meaning and being changed by the work	5

#### Theme 1: Reliving the past in the present

This theme explores how participants' own traumatic experiences resurfaced within their work. While the activation of memories could be retraumatising, it also offered opportunities for personal growth as participants confronted their fears and reshaped their narratives.

##### Subtheme 1: Retraumatised and working with triggers

The majority of participants encountered situations in their work that echoed past trauma. This subtheme provides context for the rest of the analysis by presenting the fact that working in a secure unit often comes with a level of interpersonal risk, and staff encounter violent and aggressive behaviour regularly. For staff who have experienced interpersonal abuse, such behaviour reminded them of their own abusers.

When I did first start this job, I think I was quite like I said scared to go on the wards because that experience had make made me more wary of men, it's like a domino effect (Charlotte)

For Charlotte, encounters with aggressive men in her current work created a cascade of memories and reactions (‘a domino effect’) due to their retriggering of the past. Participants reflected on the tension and apparent contradiction in choosing to work in an environment where aggression is common. Encountering reminders of their experiences triggered old memories and emotions that staff needed to manage in the moment, while also providing care to others. Rebecca described past emotions resurfacing as feeling like she was ‘a little kid again’.

It definitely kind of sets me back into the scared little kid thing and I feel like I'm out of control (Rebecca)

In this quote, Rebecca recalls being put back into a space where she feels small and vulnerable despite being in a position of relative power in her job. She also talks about feeling out of control, which seems significant in an environment in which a level of control is essential. Sara similarly experienced intrusive feelings and memories in the form of intense flashbacks, triggered by her working environment.

When he started going off in that meeting, it did sort of kind of bring some other flashbacks where I've had sort of past trauma from childhood (Sara)

As well as experiencing flashbacks of past trauma, Sara was subjected to further potentially traumatising experiences at work in the form of racism.

So this is sort of my first time meeting this patient and he was being racist (Sara)

Sara and other participants not only had to manage the impact of past trauma, but were exposed to further trauma through the system they worked in. George said that his ‘head had melted’ because of being traumatised by the things he was exposed to in his workplace. Being repeatedly exposed to violence over time had a significant impact on his mental health. George and Sara's experiences in particular suggested that at times their symptoms of trauma had been exacerbated as a result of the work that they did. George reflected on the detrimental impact his work had on his mental health.

I got burnt out and then had six months off and I got diagnosed with depression then some of the events that happened on the [name of unit] triggered some events that happened to me in the past (George)

Conversely, for Jane, although trauma symptoms were extremely unpleasant to experience, she could also recognise the benefit they presented in keeping her safe on the ward.

Things like hypervigilance, that's fantastic when you're on a secure ward with loads of quite violent dangerous offenders who are also really unwell (Jane)

It seems that although participants were wary of encountering triggers at work, a chaotic and somewhat risky environment felt normalised to them in the context of their previous experiences. It appeared as if participants were seeking out environments that felt familiar to them, and it could be that the tumultuous environment of the ward fit with their understanding and view of the world. Furthermore, as in Jane's example, it appeared to allow participants to reframe their trauma symptoms (such as hypervigilance) as a form of strength.

##### Subtheme 2: Facing fears and rewriting the script

Although encountering violence and aggression in work was understandably challenging, it presented an opportunity to face fears and create an alternative narrative (a ‘re‐writing’ of the person's life script). For example, participants faced these experiences as adults, rather than children, and in a position of relative power and control. This offered a chance for them to approach situations of aggression or abuse differently and rewrite the script, either in their own story or the stories of others. For Charlotte, she initially felt scared about going onto male wards, but doing so forced her to face her fears around men and discover things were not exactly as she expected.

I think it's just given me chance to kind of warm up to them and you know it's seeing them as actual people, because you know they are people and not just you know scary men (Charlotte)

Similar to Charlotte, Jane wondered if her experiences drew her to work with perpetrators of violence in order to better understand them and, in turn, her own abuser. In seeking that understanding, Jane could develop empathy for people despite their actions. It could be that through learning to care for patients who are both perpetrators and victims of abuse, staff can heal themselves through healing others.

I don't know whether it's something about wanting to combat that fear of people and actually recognising that people have maybe done things to me because they were suffering and actually feeling empathetic toward that (Jane)

For Jane, while there is a sense of benefit and the potential for resolution, there remains a sense of having to fight or battle (‘combat’) her past. Although for Jane there was a sense of moving forward, Sara felt her trauma was in some ways still very present. Sara had repeatedly experienced racism throughout her life to the point where she was ‘pretty much used to it’. Although this was also the case working in secure services, Sara had strong support from others, something she had sadly not experienced in the past.

I felt like scared, isolated, then I brought myself back in the moment I was like hang on I've got my team with me, nothing, nothing can happen because I've got sort of my multi‐disciplinary team with me (Sara)

In the quote above, Sara describes not just what it's like to receive support, but also to feel supported around her. Participants described past trauma, often from a position of isolation and vulnerability. For Sara, the experience of support was extremely powerful in that she was no longer in a position of vulnerability and able to rewrite her own script. Furthermore, noticing inequalities in the system prompted her to take steps to encourage positive change and, in turn, rewrite the script of those in her care.

One of the things that I highlighted to my head of department was there was a lack of cultural awareness, so I decided to come up with like a group for patients (Sara)

James found that a difficult part of his job was recognising how he might be perceived by his patients as a man. Men were often the perpetrators of patients' abuse, particularly in the female wards, and therefore, James reflected on how his attempts at care could be triggering.

James was passionate about offering an alternative narrative to the women he worked with, one in which men could be calm and empathetic.

Talking about feelings, talking about compassion as a man goes the odds of what they think, and especially if you know look like I do (James)

Like James, Mark also reflected on how managing risk on the ward could mirror difficult experiences from patients' pasts. For Mark, taking the time to explain his actions was a chance to rewrite the script for those he needed to restrain.

You've had a relationship with them, but you're here you're putting hands on them and it's always important for me afterwards when I've done those situations I will always seek out that patient afterwards and explain to him that that was not what I wanted to do (Mark)

After experiencing trauma, participants understood what it felt like to struggle with the impact of what had happened. Subsequently, many reflected on the support they have received from services or the lack thereof. Providing care to those struggling with mental health problems allowed participants to move from a position of needing care to the position of caring for others. In doing so, participants used their experiences to provide the care that they themselves would have appreciated in their times of need.

I think that's the continuation of compassionate care is advocating for that person and being like how would I want to be spoken about right now because I've not been in a forensic situation but I'm sure I've been a name in a room somewhere (Jane)

In the above quote, Jane reflects on how rewriting someone's story can be done even when they're not in the room. She talks about the power of advocating for those who are unable to do so themselves, while reflecting on a time she felt she did not have a voice.

#### Theme 2: True empathy both as gift and burden

This theme explores how participants' personal experiences of trauma influenced their capacity for empathy and connection with their patients. However, this same depth of connection often came at a personal cost, leading to emotional strain and moral tension.

##### Subtheme 1: Lived experience as a tool

Participants reflected that their experiences enabled them to identify with patients in a way others could not. Having experienced trauma themselves, participants could truly empathise with patients. While participants were clear that they felt that people who have not experienced trauma are able to understand those who have, they also felt that lived or shared experience created a deeper sense of understanding more quickly.

It's definitely made me more empathetic towards them when they're in like the crisis and having incidents and things. Like yeah, I think it's much easier for me to understand where it's coming from my life (Rebecca)

Rebecca is clear that her own experiences have allowed her to maintain understanding and connection to her clients even during intense ‘crisis’. For participants, it seems that putting yourself in someone else's shoes is always possible, even if the experiences are very different, but doing so feels more natural to those who have walked a closer path. Not only can lived experience be used to facilitate understanding, but using it in a positive way allowed Jane to find meaning and purpose in her own trauma.

I obviously don't tell my patients what's happened to me but I find it's actually really helpful, and I'm not, I'm not grateful it happened I'm not happy, none of that but it is nice to have for it to have a use and for it to have a purpose (Jane)

Jane was able to change the meaning of her experiences by making use of them at work. Such experiences became valuable, offered power and began to serve a function like a ‘tool in my tool kit’.

Sarah also used her experiences to support both patients and colleagues, helping her identify warning signs of the same difficulties in others.

I'm able to sort of reflect on it and sort of use that experience as well when I work with patients and colleagues as well (Sara)

George saw his experiences as a means to connect to his patients, particularly those that were raised in the care system. While George empathised with others from a similar background, he emphasised that he himself had learned to ‘grow up and evolve’ and sought to help his patients to do the same.

a lot of these lads also came through care homes and foster homes so my foot in the door so to speak, was there because I could relate, I've been there and done it (George)

Mark similarly felt that his past experience of care shaped his own caregiving, helping him to appreciate his patients' heightened need for compassion and understanding.

the way I was looked after during that time of my life by health professionals if you like made me realise that it's, I don't know, it makes me realise that you need to be compassionate as well (Mark)

James also felt that his experiences were important to the work he did, particularly in being able to give sincere empathy. He felt his trauma not only strengthened his compassion but also protected him from burnout and compassion fatigue.

it's armour plated my compassion because I've lived it you know and I can genuinely give that empathy now (James)

James used his trauma in the work he did and allowed it to become a strength; his trauma became armour around his compassion, making it unshakable.

##### Subtheme 2: Cost of empathy and connection

Although participants found purpose in their experience, this also came at a cost. Having a deeper understanding of patients' feelings and experiences triggered intense emotions and a feeling that they were carrying the experiences of patients as well as their own. In connecting with patients on a deeper level, staff opened themselves up to resonating with the intense emotions of their patients. James described how true empathy could lead to absorbing parts of patients' experiences.

Learning about personal tragedy from the patients that we work with and because we try and empathise as much as we can there's bits of that that you take on to yourself (James)

Identifying with patients in this way offered a means to establish rapport and interpersonal connection but became problematic when staff overidentified with their patients. Charlotte spoke about the difficult emotions that arose from meeting patients she saw as similar to herself.

a guy that's not that much older than me and he could potentially spend the rest of his life in a place like this and it does it genuinely upsets you (Charlotte)

For Rebecca, the cost of true empathy and connection could at times be her own mental well‐being. Caring for those with severe and enduring mental health difficulties seemed to be a balance between putting yourself in their shoes and not allowing yourself to be pulled into the same headspace.

it's just like sometimes you start to see their point of view which is really dark because you're like actually I can get it, so it kind of takes you to a bit of darker place (Rebecca)

Rebecca found meaning and satisfaction in her work but reflected on the toll it could take on her outside of work.

I've gotten home from shifts that have been very, very difficult and kind of broken down and been crying my eyes out (Rebecca)

Due to the emotional toll that this level of care takes, Mark felt that taking time away from work was the only way to prevent himself from burning out.

if you can't switch off from work when you come home then you're gonna burn yourself out quick, so it's important to be able to enjoy the time that you're not in work (Mark)

As well as building strong therapeutic relationships with patients, staff in secure services are often put in the position of limiting a person's access to amenities or having to physically restrain patients. Some participants felt that this was a difficult position to take, particularly when they could strongly empathise with that person.

My first thing was just I wanted to hug him but we had to put him in in the seclusion area to keep himself safe (George)

Needing to switch between a position of care and compassion to a position of authority and enforcement had the potential to leave staff with a moral dilemma. There seemed to be internal conflict in staff between understanding restraint and restriction as necessary to keep everyone safe, while feeling pulled towards providing more gentle, compassionate care.

#### Theme 3: Identity: Giving and taking parts of your self

This theme explores how participants' self‐identity was closely connected to their professional roles and highlights the complexity involved in being authentic at work. While such authenticity created personal meaning and fostered compassion for others, it also required careful navigation of boundaries to maintain safety and well‐being.

##### Subtheme 1: The cost and benefit of giving your authentic self to your work

Many participants spoke about their work, which gave them purpose and was an integral part of who they were. Participants often discussed their identity in relation to their job and how the two were intertwined. Many participants felt that an important part of their work was putting their authentic selves into their interactions with patients. Giving parts of themselves seemed to be beneficial in gaining patients' trust and providing care with genuine sincerity. However, this can also be challenging in a forensic setting while trying to manage risk and maintain professional boundaries.

Rebecca felt that the relationship between who she was and her work was bi‐directional. She felt that her identity as a person influenced how she performed her work and what she brought to the workplace. However, she also felt that the work she did had a profound impact on who she was as a person.

Every part of who you are in mental health affects your home life and everything about who you are outside of the home affects how you behave in mental health (Rebecca)

Rebecca's quote suggests that her work and identity were not only related but were enmeshed and impossible to separate.

Charlotte spoke very differently about her identity in relation to work compared with other participants. For Charlotte, trying to hold boundaries and separate parts of herself was a way of coping.

I just put up like a wall between my personal life and my work life I think anybody in that position … it's going to be a bit daunting, but you just get used to it very quickly, like I said you put up those walls, it's very different outside of work (Charlotte)

Bringing parts of oneself openly to work has the potential to leave staff feeling vulnerable, especially in an environment where risk to staff is ever‐present. For Charlotte, it may have been more comfortable to keep certain aspects of herself protected. This could feel less exposing but could also limit the impact of taking on patients' emotions and experiences to protect her home life. This is supported by some of Charlotte's later reflections, which suggest that holding rigid boundaries can be challenging, especially when forming connections with patients and when faced with their emotionally charged stories.

Having that block was between your professionalism and your emotions as well you know like I said I feel really sorry for these young people who are in this type of facility, you have to also be professional (Charlotte)

Other participants reflected on the tension between bringing your true self to the work you do versus keeping yourself safe, both physically and emotionally. James emphasised the importance of judgement and experience in finding that delicate balance in such a challenging environment.

You definitely need to judge it and this is very important because as you're very aware if you're open you leave yourself open (James)

Despite recognising the vulnerability that came with bringing his identity into his work, James still felt that this was a core part of the role he did and the practitioner he was.

I am me, that is my entire job, there's not artifice there's just I come in and I am me (James)

This suggests that, like Rebecca, his identity was enmeshed with his work, and both formed a part of the other. And like Rebecca, James found he was only able to present a limited ‘version’ of himself. Although there was a benefit to doing so, there is also a cost to walking such a fine tightrope.

I am the version of me here and sometimes that mask can slip and that's when we tend to find issue (James)

Although there is a balance to be found with how much of themselves staff brought to their work, there seemed to be an emphasis on honesty and sincerity. For Jane, being authentic and genuine was essential. She felt that bringing herself and who she is to the role was the only option she could envision.

I can't really think of any other way that I would be, do you know what I mean I can't imagine divorcing myself from that way of being (Jane)

Participants were careful to explain that bringing their authentic selves to the work they did came at a cost and needed to be done thoughtfully and with caution. However, it seemed that for some, there was an inevitability to their identity becoming intertwined with the work they did. Even participants who believed in holding firm boundaries between themselves and their work found this was not completely achievable.

##### Subtheme 2: Gaining personal meaning and being changed by the work

As participants discussed their identity, they also reflected on how the work they did had changed them. Many participants were drawn to working in secure services to help others, giving them a sense of meaning and purpose. This could result in positive changes such as increased compassion for others, a sense of being part of something bigger than yourself and increased self‐belief. However, participants could also be negatively affected by their work, either through taking on too much from their patients, burnout or prolonged exposure to violence. For Rebecca, working in secure services altered her self‐perception and the beliefs she held about herself and her abilities.

Makes you feel really nice and good about what you're doing and your job like no actually I'm not shit, I can do this, I can be helpful I've got it like it just gives you a little boost to your ego (Rebecca)

It seems that finding meaning and validation through work had the power to override deeply held negative beliefs that had been ingrained since childhood. Conversely, for George, his work perpetuated his exposure to violence to the point where it became normalised.

I'm willing to walk into violence I think that's the negative impact, really it's a big negative because I don't think twice about jumping into shit now (George)

This change meant he no longer held the same regard for his own safety, especially when he felt compelled to protect others. Despite this reflection, George was also incredibly positive about the work he did and the experiences it had brought him, describing it as ‘the best job in the world’. Working in secure services offered staff a chance for personal growth through rising to challenges and persevering in the face of them. Furthermore, for James, caring for others and experiencing difficulties led him to learn to care for himself.

it's made me take my mental health more seriously like I was the classic puritan worker very proud of the fact that well I've not had a day off sick in seven years even though I was there crying (James)

Additionally, managing challenging situations at work also seems to facilitate bonding and compassion within the team.

We all lookout for each other we all are there more so than say we were previously and that for me a driver is from what I've learned through this job (James)

Similarly, Mark described finding a sense of purpose and a path in life he might not have otherwise considered if it were not for the things he experienced in his work.

I am on the path to going and doing my nurse training and I would say it's partly because, if not mainly because of that experience that I endured within the first six or seven months of working in the job (Mark)

Finally, Jane spoke about finding meaning in her work through gaining patients' trust and respect. She found the experience of being the first person someone opened up to a ‘privileged position to be in’ and felt proud that patients could share with her. Working in secure services is extremely challenging yet powerful work. Most found that the personal meaning and purpose that grew from the work was worth the struggles that came with it.

## DISCUSSION

To our knowledge, this is the first study exploring how personal trauma impacts how staff in forensic mental health services give compassionate care. Three main themes were identified from participant interviews: reliving the past in the present, true empathy both as a gift and a burden, and the costs and benefits of gaining personal meaning and being changed by the work. Participants reported several ways trauma impacted them in their work and their personal lives, both positively and negatively. Overall, a common thread that came through in all interviews was that trauma was not a barrier to compassion, but rather a facilitator.

Previous research suggested that forensic staff are exposed to a variety of distressing events, such as self‐harm and patient suicide (Mistry et al., 2022; Stewart et al., 2012). The findings from this study support this research as participants reported being regularly exposed to extremely distressing events throughout their careers. Participants in this study also reflected on the challenges that came with caring for violent patients and how this could move them from a compassionate mindset to a place of threat. This could result in staff feeling defensive or avoidant around particular patients. This is in line with past evidence that staff may struggle to remain compassionate and caring when feeling threatened by patients in forensic settings (Adriaenssens et al., 2012; Hammarström et al., 2020; Purpora et al., 2015). Hubbard et al. (2017) found that personal experience of trauma was a risk factor for secondary trauma in HCPs. Participants in the Hubbard et al. (2017) study reflected on the difficult emotions that were brought up for them while working with someone who had experienced similar trauma, which would support those findings. One consequence of trauma can be ‘reliving’ experiences or experiencing flashbacks of the traumatic experience. Both involve involuntarily remembering parts of traumatic experiences and can be triggered by specific reminders that relate in some way to the trauma (Brewin & Holmes, 2003). It may be that for participants, some behaviours exhibited by patients could be related to their past trauma and trigger difficult memories.

As discussed in the introduction, the evidence on the impact of trauma on HCPs' compassion is mixed. Some studies suggest that HCPs' experiences of trauma can lead to compassion fatigue and impair patient care (Hensel et al., 2015; La Mott & Martin, 2019; Lakioti et al., 2020; Pereira et al., 2017; Somoray et al., 2017). However, the findings of this study suggest the opposite may be true. Participants in this study reported that their experiences of trauma resulted in deeper empathy and strengthened their compassion for others. Participants felt that their own experiences of trauma allowed them to better understand the struggles of those they worked with, which is consistent with the findings of Day et al. (2017). Furthermore, although there is a suggestion that those working in forensic settings struggle to remain empathetic and provide compassionate care (Sturzu et al., 2019), the reality appears to be more complex. For example, although participants did reflect on times where compassion felt difficult, overwhelmingly, compassion was a motivating factor behind the work they did. Additionally, personal experiences of suffering have previously been found to facilitate compassion in HCPs as they create a deeper understanding (Singh et al., 2018). This is very much in line with the findings of this study as participants used their own experiences to create a deeper understanding of their patients.

Although the focus of this study was compassion, it is also important to consider the interplay of closely related concepts, such as empathy. Although the terms compassion and empathy are often used interchangeably, they are distinct concepts (Jeffrey, 2016). Whereas compassion requires recognising the suffering of others or oneself, empathy has been conceptualised as the mirroring of that emotion (Peters & Calvo, 2014). Compassion is described as feeling for, whereas empathy is described as feeling with (Singer & Klimecki, 2014). Compassion can also be understood as a motive linked to caregiving, whereas empathy can be deemed a competency or skill that can support this motive (Gilbert, 2020). Participants in this study described identifying with patients and sharing their emotions, suggesting the presence of both empathy and compassion. Although compassion satisfaction seems to be high within this population, the role of empathy needs to be further considered as higher empathy can be a risk factor for burnout (Pirelli et al., 2020).

Although the majority of research focuses on the negative consequences of trauma, there has been growing interest in post‐traumatic growth. Post‐traumatic growth is the concept which acknowledges the negative consequences of trauma but also recognises that there can be positive consequences as people find personal growth through adversity (Lindstrom & Triplett, 2010). This study captured participant post‐traumatic growth in the form of strengthened empathy and compassion. For example, participants could rewrite the script, both for themselves and their patients, by providing compassionate care for others. This is reminiscent of the concept of ‘the wounded healer’ whereby a ‘healer's wounds can be curative for those they work with (Zerubavel & Wright, 2012). This concept recognises the benefits that can come from HCPs using personal experiences in their work, as do the findings of this study.

### Implications for clinical practice

The results of this study have the potential to inform both patient care and staff well‐being.

Although staff experiences of trauma seem to deepen compassion and understanding in the long term, there are some negative effects on staff. For example, staff who have experienced trauma perceive themselves as connecting with patients on a deeper level, leading to more intense feelings in the therapeutic relationship. Additionally, working with aggressive patients or patients with similar experiences can trigger trauma symptoms for staff.

Staff may benefit from a reflective space to reflect on intense therapeutic relationships and how their experiences impact their work. Reflective practice groups (RPG) can offer staff a safe reflective space, which research suggests HCPs find to be beneficial (Fisher et al., 2015). Staff working in low secure services reported that RPG offered an opportunity to gain self‐awareness and to ‘off‐load’ (Tulley, 2021). RPG may already be in place in some forensic settings; however, the importance of these practices should be reiterated and made available to as many staff as possible. Having a space, such as an RPG, could help staff increase their awareness of how their own experiences are impacting their work and allow them to express emotions that might otherwise go unaddressed. This is supported by evidence that high‐quality reflective practice can mitigate the impact of vicarious trauma in caring professions (Hazen et al., 2020). In addition to HCPs, it may be beneficial for staff to engage in individual therapy to provide a safe, private space to address traumatic experiences.

Trauma‐informed care (TIC) involves considering the role of trauma in someone's life and how one might minimise the impact of that traumatic stress (Wilson et al., 2013). There is a high prevalence of trauma and PTSD in forensic mental health patients (Bianchini et al., 2022; Spitzer et al., 2001), meaning TIC is even more important in a forensic setting. The literature specifies that in order to truly integrate TIC into forensic mental health practice, this would involve a shift in everyday working and thinking, as well as a shift in the culture of an organisation (Hiett‐Davies, 2022). This shift in culture and everyday working towards TIC could be beneficial not only for patients in forensic settings but also for staff.

Ultimately, this study reaffirms the significance of lived experience and the profound impact of shared experiences. This has previously been highlighted in the literature as well as the struggle of disclosing experiences while maintaining appropriate boundaries (Lovell et al., 2020; Rachwal & Gredecki, 2024). If staff are considering disclosing shared experiences with patients, this should be carefully considered to ensure appropriate boundaries are held in place. The Sharing Lived Experiences Framework (Dunlop et al., 2021) provides a clear framework that can be used to assist mental health practitioners who are considering disclosing their lived experiences.

### Strengths, limitations and implications for future research

This study presents a novel contribution to the evidence base as there is currently no research examining forensic staff experiences of personal trauma and its relation to compassion. The researchers therefore chose a methodology which allows the focus to remain on individuals' stories of their lived experience (Smith, 2017). Using IPA allowed researchers to collect rich data, but also resulted in a smaller sample being recruited. Future studies may wish to explore this topic with a larger sample to understand whether the experiences of participants in the current study are unique to them. A key limitation of this study is that it relies on staff volunteering to speak about their traumatic experiences. This naturally introduces a sampling bias, as staff who are struggling with trauma symptoms may not feel ready to talk about them. However, this is a limitation that cannot easily be overcome.

Past research investigating the experiences of forensic staff has mainly been focused on nurses. This study provides a valuable and novel addition to the literature, as a range of HCPs were recruited. The sample was diverse in terms of job role, as well as their traumatic experience(s). This could be considered a strength, as the results speak to the impact of many forms of trauma. However, Sara (for example) spoke about her trauma in relation to experiencing racism. Other participants experienced things that they found triggering or echoed their trauma in some way. Conversely, Sara was repeatedly exposed to racism to the point where she felt it was normal and expected. Therefore, further research on the impact of racism and racial trauma within this population would be valuable. This seems especially important as institutional racism in mental health systems is a documented issue (Fernando, 2017). Additionally, future research could build on these findings by exploring what support staff who have experienced trauma would benefit from, especially in relation to strategies to support staff well‐being and maintain healthy boundaries.

## CONCLUSIONS

The findings of this study suggest that staff feel that trauma can have some negative impacts on their work. However, overwhelmingly, the participants in this study highlighted the ways in which their experiences could be utilised as a tool in their work. Although compassion comes at a cost, it seems to motivate staff in forensic settings to persevere in challenging circumstances (supporting the notion that the motivation to care remains despite various obstacles). This study suggests that forensic staff's experiences of trauma can facilitate compassion in the long term via post‐traumatic growth.

## AUTHOR CONTRIBUTIONS

**Bethan Owen:** Conceptualization; writing – original draft; methodology; writing – review and editing; formal analysis. **Tobyn Bell:** Writing – review and editing; methodology; supervision. **Katherine Berry:** Writing – review and editing; project administration; supervision. **Brendan J. Dunlop:** Conceptualization; writing – review and editing; supervision; project administration.

## CONFLICT OF INTEREST STATEMENT

The authors declare no conflicts of interest.

## Data Availability

The data that support the findings are not publicly available due to privacy or ethical restrictions.
